# Novel device to prevent droplets in bronchoscopy during the SARS‐CoV‐2 pandemic

**DOI:** 10.1111/1759-7714.13729

**Published:** 2020-11-04

**Authors:** Keigo Uchimura, Kei Yamasaki, Toshinori Kawanami, Hideki Kanda, Hideki Sakakibara, Kazuhiro Yatera

**Affiliations:** ^1^ Department of Respiratory Medicine University of Occupational and Environmental Health, Japan Kitakyushu Fukuoka Japan

## Abstract

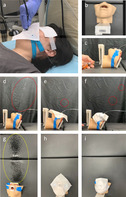

We modified the method of protection to reduce the exposure of health‐care workers (droplets) without restricting operability during bronchoscopy. Our method is inexpensive, simple, utilizes disposable materials and prevents interpatient infections. Its routine use during transoral bronchoscopy may be considered due to its simplicity.

Coronavirus disease (COVID‐19) is caused by severe acute respiratory syndrome coronavirus 2 (SARS‐CoV‐2), which spreads through droplets, aerosols, and contact.^1^ Flexible bronchoscopy (FB) is a diagnostic and therapeutic modality for respiratory diseases but has a high risk of viral transmission via droplets and aerosols. Therefore, reducing healthcare workers' (HCWs) exposure is important. Bronchoscopy guidelines recommended postponement, but, if essential, HCWs should wear full personal protective equipment (FPPE).^2^, ^3^, ^4^ Various measures have been reported to prevent splashing via reflex vomiting, sneezing, and coughing during esophagogastroduodenoscopy.^5^ However, there are insufficient reports on FB despite the higher risk of cough reflexes than in esophagogastroduodenoscopy. Advanced bronchoscopic procedures, such as endobronchial ultrasound‐guided transbronchial needle aspiration, require an operator/assistant to hold the scope near the patient's mouth.

We modified the method of protection to reduce HCWs' droplet exposure without restricting operability (Fig 1a). We propose the use of a mouthpiece with tips for a fixing belt (HZ712804, U.S. Endoscopy Group Inc., OH, USA) and nonwoven fabric (NWF) (PROWIPE, DAIO PAPER Co, Tokyo, Japan) for daily use at each facility. The NWF can be firmly fixed to the mouthpiece (Fig 2). FB, including oral suction, can be conveniently performed through the central X‐shaped cut of the NWF. Bronchoscopy guidelines suggest oral insertion through a small incision in a standard surgical mask (Fig 1f,i) or nasal insertion.^2^, ^3^, ^4^ However, unlike the NWF, the mask is fixed to the patient's ears, so the incision in the mask and mouthpiece may misalign as patients move or during FB removal/reinsertion. Transnasal FB is not popular in Japan because of the limited operability. In our method, the fixed NWF does not shift from the mouthpiece. The COVID‐19 pandemic has depleted medical resources such as surgical masks. To examine the reduction of droplets, we compared the number of droplets with no equipment, NWF, and surgical mask using a simulated patient (Fig 1b‐1i). NWF and surgical masks significantly reduced splashes equally (Fig 1d‐1i). Our method is inexpensive, simple, utilizes disposable materials, and prevents interpatient infections. This method requires HCWs to use FPPE, but its routine use during transoral FB may be considered due to its simplicity.

**Figure 1:**
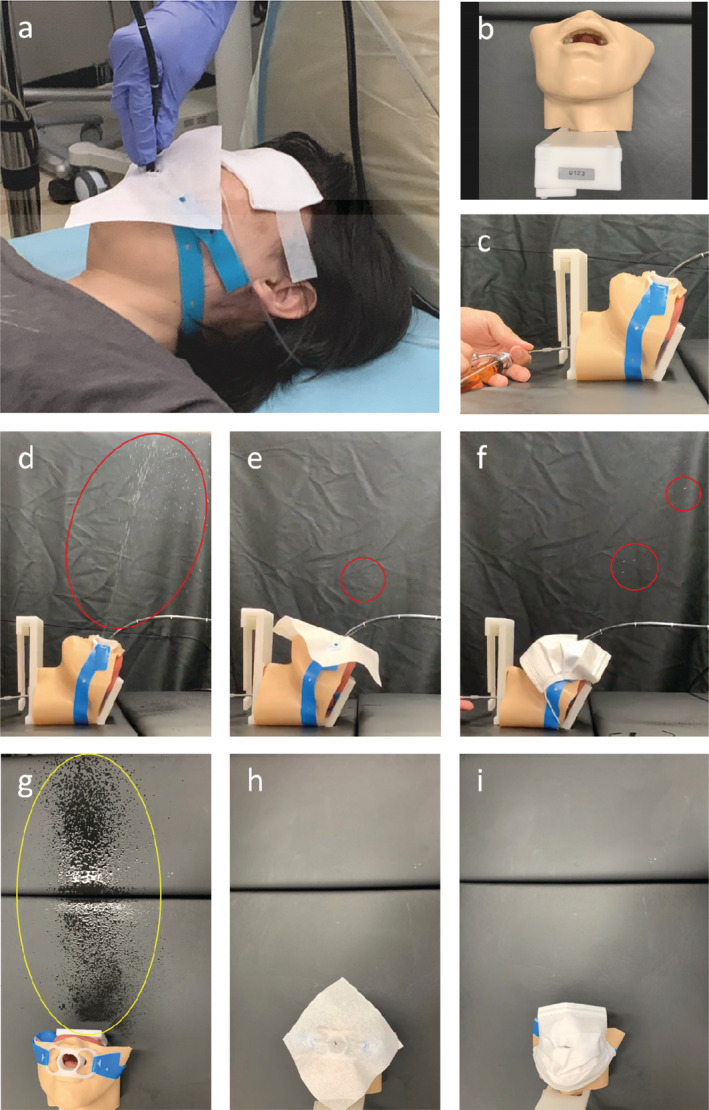
Bronchoscopy with a nonwoven fabric (NWF) and comparison of the number of splashes using a simulated artificial patient model. (**a**) Bronchoscopy with a NWF fixed to the mouthpiece. The nose and mouth are well covered because the NWF blocks droplets from the patients. Bronchoscopy, including oral suction and nasal oxygen administration, can be performed conveniently through a central X‐shaped cut in the NWF. (**b**) A bronchoscopy training model (LM‐099B, KOKEN, Tokyo, Japan) used as a simulated artificial patient model. (**c**) To artificially create the splashes out of the mouth, a Jackson‐type spray (Matsuyoshi & CO., Ltd., Tokyo, Japan) was placed from the subglottic region to the oral cavity in the model, and 3 mL of saline was sprayed vertically with only the mouthpiece (**d** and **g**), NWF (PROWIPE, DAIO PAPER Co, Tokyo, Japan, **e** and **h**), and with a surgical mask (2827J, 3M Japan Limited, Tokyo, Japan, **f** and **i**). (**d**) A large number of droplets (red circle) were scattered with only the mouthpiece. (**e** and **f**) Droplets (red circles) were slightly visible with the NWF (**e**) and surgical mask (**f**), but were clearly reduced with the NWF (**e**). (**g**) Extensive contamination (yellow circle) was confirmed with only the mouthpiece after spraying. The area around the model did not get wet from splashes after spraying with the NWF (**h**) and the surgical mask (**i**).

**Figure 2:**
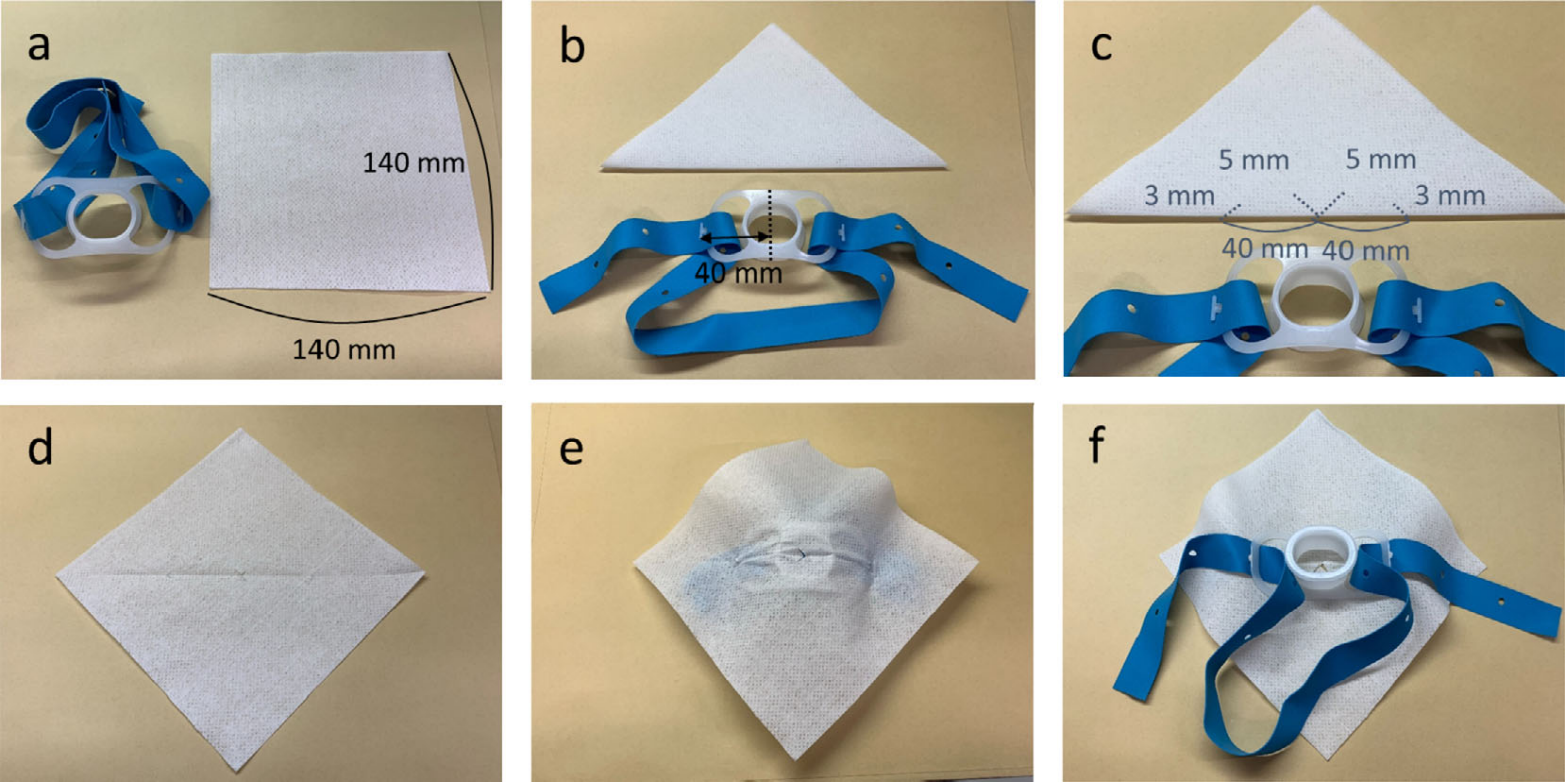
Preparation of the nonwoven fabric (NWF) and mouthpiece. (**a**) Prepare a mouthpiece with tips for the fixing belt. Cut the NWF into 140 mm squares. (**b**) Fold the NWF half diagonally and measure the length from the center of the mouthpiece to the tip (depending on the prepared mouthpiece). (**c**) Make two 5 mm cuts at right angles in the middle of the bottom of the NWF. Make 3 mm notches at the left and right sides of the measured length. (**d**) The open NWF has an X‐shaped cut in the center and V‐shaped cuts on the left and right. (**e**) Front side of the finished product. Complete by covering the mouthpiece with the NWF and fixing the left and right V‐shaped notches to the tips of the mouthpiece. (**f**) Reverse side of the finished product.

## Disclosure

The authors declare that they have no competing interests.
